# Nicotine-enhanced stemness and epithelial-mesenchymal transition of human umbilical cord mesenchymal stem cells promote tumor formation and growth in nude mice

**DOI:** 10.18632/oncotarget.22712

**Published:** 2017-11-27

**Authors:** Tao Li, Jun Zhang, Jiahui Zhang, Nannan Zhang, Yang Zeng, Shengnan Tang, Zehua Tao, Xiying Qu, Jue Jia, Wei Zhu, Xiaochun Sun, Huabiao Chen

**Affiliations:** ^1^ School of Medicine, Jiangsu University, Zhenjiang, Jiangsu 212001, P.R. China; ^2^ Department of Laboratory, The Affiliated Hospital of Yangzhou University, Yangzhou, Jiangsu 225001, P.R. China; ^3^ Vaccine and Immunotherapy Center, Massachusetts General Hospital, Harvard Medical School, Boston, MA 02114, USA; ^4^ The Affiliated Hospital of Jiangsu University, Zhenjiang, Jiangsu 212001, P.R. China

**Keywords:** cancer, nicotine, mesenchymal stem cells, stemness, EMT

## Abstract

Cigarette smoking is a well-known risk factor in the development and progression of malignant diseases. Nicotine, the major constituent in cigarette smoke, has also shown negative effects on stem cells. Mesenchymal stem cells (MSCs) have been widely demonstrated to migrate into tumors and play key roles in cancer progression. However, the mechanisms by which nicotine impacts MSCs and tumorigenesis of lung cancer are still undetermined. In this study we investigated the effects of nicotine on human umbilical cord mesenchymal stem cells (hUC-MSCs) and the impacts of nicotine-treated hUC-MSCs on tumor formation and progression. We found that nicotine has a toxic effect on hUC-MSCs and changes the morphology, inhibits proliferation and promotes apoptosis of hUC-MSCs in a dose-dependent manner. Nicotine-treated hUC-MSCs produce higher level of IL-6. Moreover, nicotine promotes migration, stemness and epithelial-mesenchymal transition (EMT) of hUC-MSCs by inhibiting E-cadherin expression and upregulating mesenchymal markers such as N-cadherin and Vimentin, leading to the induction of stem cell markers Sox2, Nanog, Sall4, Oct4 and CD44. Migration and proliferation of non-small cell lung cancer A549 cells and breast cancer MCF-7 cells are promoted after their coculture with nicotine-treated hUC-MSCs in a cell-cell contact-independent manner. Furthermore, nicotine-treated hUC-MSCs promote tumor formation and growth of A549 cells in nude mice. These studies demonstrated that the enhanced stemness and EMT of hUC-MSCs induced by nicotine are critical for the development of tobacco-related cancers.

## INTRODUCTION

Cigarette smoking is a well-established risk factor in many diseases in the adult population, including cardiovascular diseases and pulmonary diseases, such as lung cancer [1]. Non-small cell lung cancer (NSCLC) accounts for about 80% of all lung cancers [2]. Tumor metastasis, which is often observed in patients who are newly diagnosed of lung cancer, is considered a crucial factor contributing to the recurrence risk, high mortality and poor prognosis of lung cancer [2]. The prevalence of cigarette smoking among adult population is still high in the world [3, 4]. Cigarette smoke is complex mixture containing more than 4,000 chemicals, including nicotine, aromatic hydrocarbons, aldehydes, hydrogen cyanide, and so forth [5]. Nicotine is the major chemical component in cigarette smoke in the form of acid salts, which are absorbed in the lungs [6] and considered as a determinant of addiction to cigarette smoking [7]. In addition, previous studies have showed that nicotine promotes proliferation, invasion and angiogenesis and inhibits apoptosis in cancer cells [8–10]. Nicotine has been demonstrated to negatively affect stem cells [11]. However, the effects of nicotine on hUC-MSCs remain largely unexplored.

MSCs are multipotent stem cells, which can differentiate into multiple cell types *in vitro*, such as osteoblasts, adipocytes, myoblasts and chondrocytes [12–15]. Bone morphogenetic proteins (*BMPs*) belong to the transforming growth factor–β superfamily and play a key role in postnatal bone formation [16]. They have a remarkable ability to induce cartilage and bone formation from MSCs. Bone morphogenetic proteins 3 (*BMP-3*) is an important member of the *BMPs*. MSCs have been shown to differentiate into an osteogenic lineage as indicated by significant increases of *BMP-3* level [17]. Peroxisome proliferator-activated receptors (*PPARs*) are well known for their control over lipid and glucose metabolism and *PPARγ-2* is associated with adipose tissue formation [18, 19]. MSCs are initially isolated from bone marrow and reported to exist in many organs and tissues of body, including umbilical cord [20–23], umbilical cord blood [24, 25], and adipose tissue [26, 27]. However, it is very difficult to isolate MSCs from human bone marrow and the proliferative and multilineage differentiation potentials of bone marrow-derived MSCs gradually decrease with aging [28]. Nevertheless, umbilical cord collection is convenient and is not associated with any ethical or legal issue [29].

MSCs are able to migrate to the site of tumor and play a key role in cancer progression but the underlying mechanisms remain largely unknown. Previous studies have demonstrated that MSCs promote tumor cell growth and metastasis [30, 31], while other studies have indicated that MSCs display intrinsic anticancer activities [32–34]. This discrepancy requires further investigation. Cancer stem cells (CSCs), or called as cancer cells with stem cell-like properties, are pluripotent cells that can self-renew and differentiate into multiple cell types [35]. Cancers are maintained by subpopulation of CSCs in aspect of tumor growth, tumor heterogeneity and metastatic dissemination [36, 37]. CSCs also exhibit resistance to chemotherapy and radiotherapy in a variety of cancers [38]. Previous studies have indicated that stem cells in breast and colon cancer may increase the properties of CSCs [39, 40] and acquisition of stemness and EMT is a crucial process in breast cancer invasion [41, 42]. Whether nicotine directly impacts hUC-MSCs and then nicotine-treated hUC-MSCs affect tumor formation and progression remains unclear. In this study we investigated the effects of nicotine on hUC-MSCs and then the effects of nicotine-treated hUC-MSCs on tumor formation and progression of A549 lung cancer. Our data provided a possible mechanistic explanation for smoking-related cancers. In addition, the effects of nicotine-treated hUC-MSCs on breast cancer MCF-7 cells were also investigated.

## RESULTS

### HUC-MSCs have the ability of multilineage differentiation

**Figure 1:**
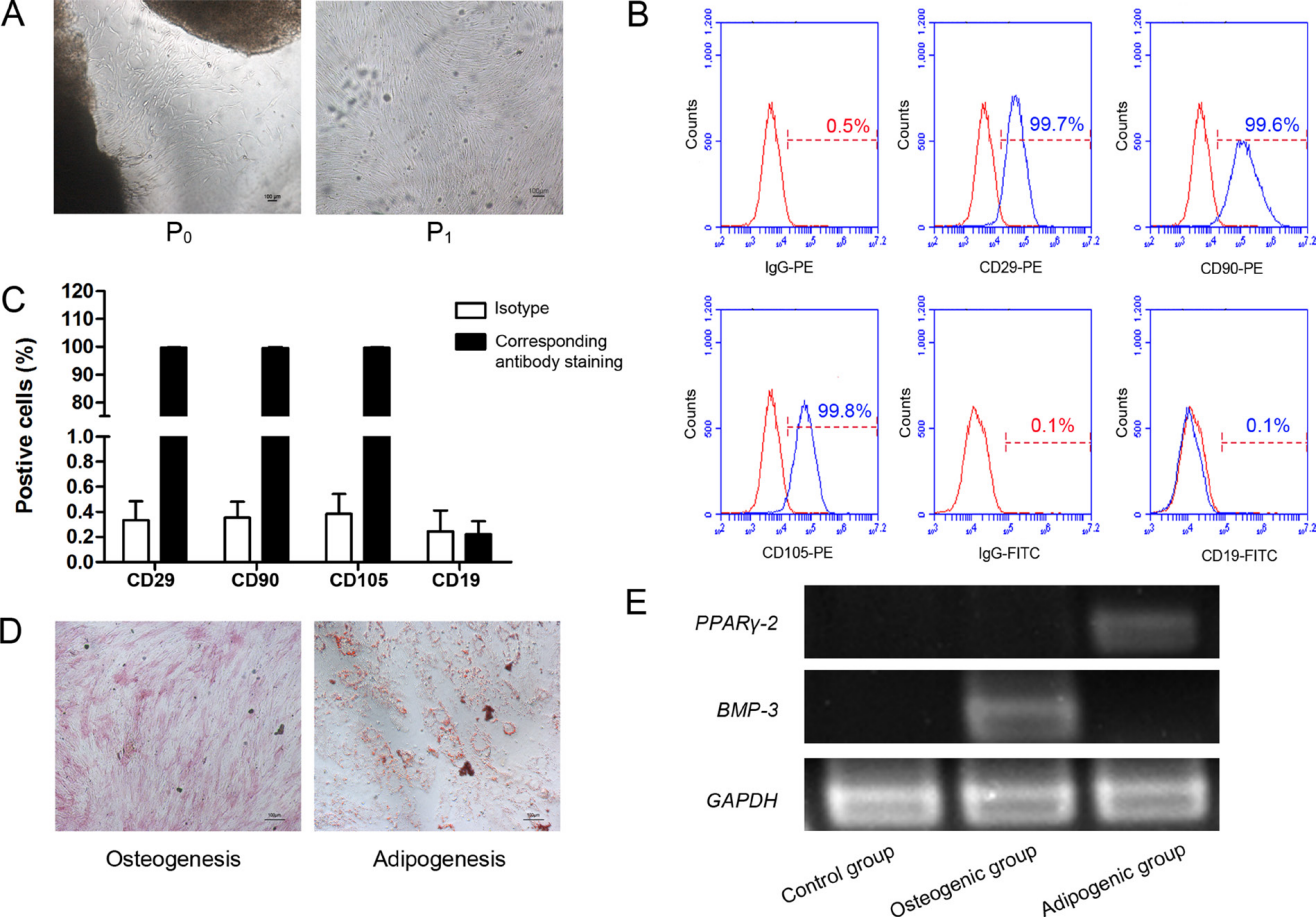
Characterization of hUC-MSCs (**A**) The cells presented polygonal, spindly and fibroblast-like. Magnifications: 40×. Scale bar: 100 μm. P, passage. (**B**) Representative histograms of hUC-MSC surface expression of CD29, CD90, CD105 and CD19, as assessed by flow cytometry. HUC-MSCs were positive for CD29, CD90 and CD105, but negative for CD19. HUC-MSCs: human umbilical cord mesenchymal stem cells; CD: cluster of differentiation; IgG: immunoglobulin G; PE: phycoerythrin; FITC: fluorescein isothiocyanate. (**C)** Quantitation of B. (**D)** HUC-MSCs were differentiated into adipocytes for 21 days. Fat accumulation was visualized by Oil Red O staining. HUC-MSCs were differentiated into osteoblasts for 14 days. Osteogenic differentiation was visualized by ALP staining (Magnification: 100×, Scale bar: 100 μm). (**E**) The expression of genes in osteogenic differentiation and adipogenic differentiation of hUC-MSC. *BMP-3* mRNA level were significantly higher compared to adipogenic group and *PPARγ-2* mRNA level were significantly higher compared to osteogenic group. *BMP-3*: Bone morphogenetic protein; *PPARγ-2*: peroxisome proliferator-activated receptors γ-2.

After 10 days of culture, the cells displayed a polygonal, spindly and fibroblast-like morphology and began to form colonies (Figure 1A). Endothelial progenitor cells were gradually eliminated after multiple medium replacements and PBS washing. Consistent with known MSC phenotypes, passage 3 cells highly expressed MSCs markers CD29 (99.7%), CD90 (99.6%), and CD105 (99.8%), while low expressed B lymphocyte surface markers CD19 (0.1%) as shown in Figure 1B, 1C. After 2 or 3 weeks in culture in the specific medium, the cells were capable of differentiating into osteocytes and adipocytes, as shown by positive staining of ALP and Oil Red O (Figure 1D), strongly suggesting that the cells have the multilineage differentiation potential. To further confirm this, expression of osteogenic and adipocyte markers were examined. *BMP-3* mRNA level was significantly higher and *PPARγ-2* mRNA level was significantly lower in osteogenic group compared to adipogenic group (Figure 1E). These data indicated that we efficiently generated hUC-MSCs which were used in the following studies.

### Nicotine treatment affects viability of hUC-MSCs in a dose and time-dependent manner

**Figure 2:**
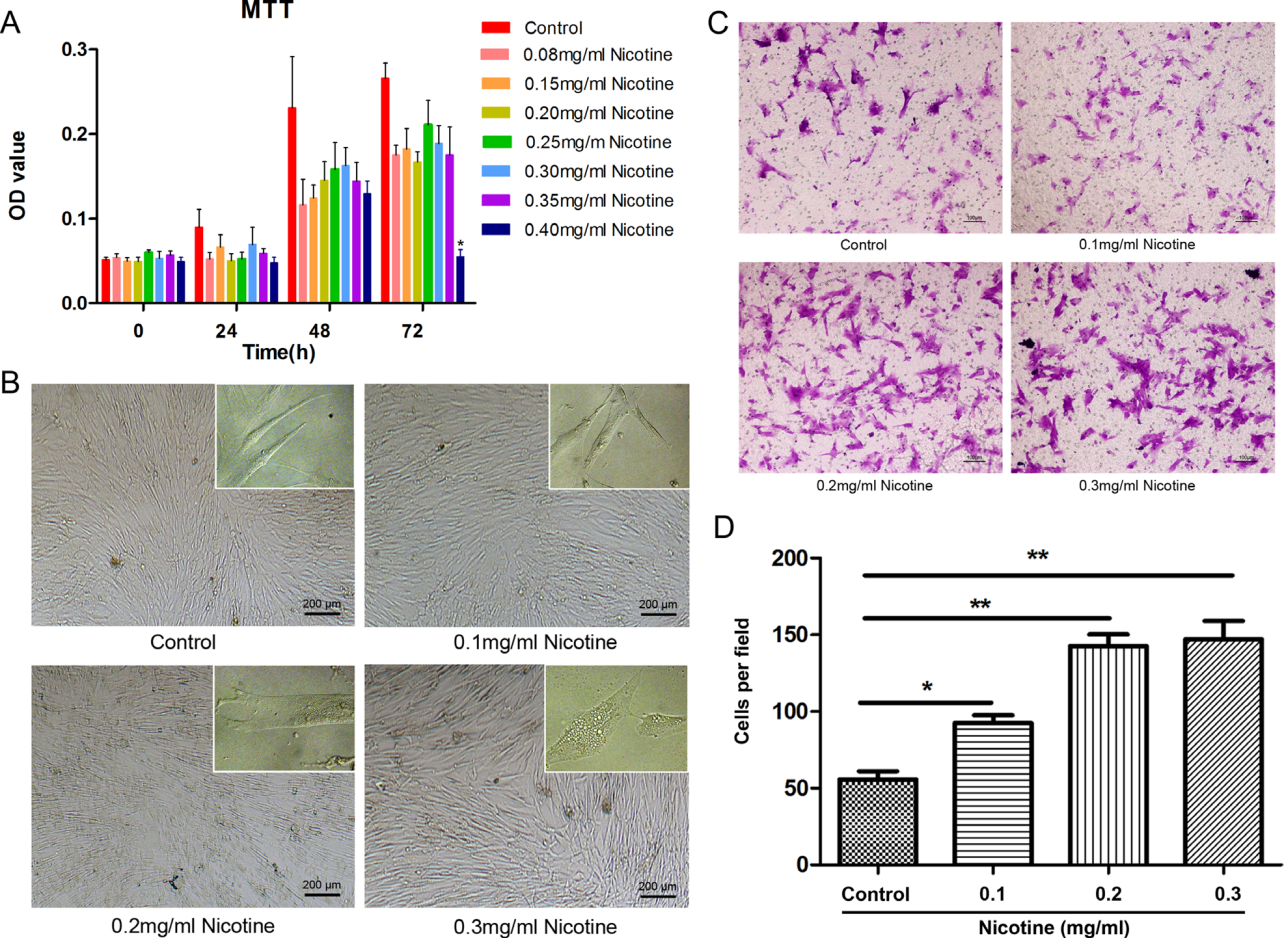
Effect of nicotine on cell viability and migration (**A)** All cells were incubated with various concentrations of nicotine (0.08 to 0.4 mg/ml) for 24 to 72 hours and cell viability was determined by MTT assay. Cell viability of hUC-MSCs was not significantly impaired until up to concentration of 0.4 mg/ml. MTT: 3-(4,5-dimethylthiazol-2-yl-)-2,5-diphenyl tetrazolium bromide. (**B)** The morphology of hUC-MSCs changed in a dose-dependent manner of nicotine which ranges from 0 up to the concentration of 0.3 mg/ml. (**C)** Representative images of transwell migration assay. Transwell migration analysis showed the number of migrated treated hUC-MSCs were more than non-treated cells. Magnification: 100×, Scale bar: 100 μm. (**D)** Histogram of the number of migrated hUC-MSCs. Magnification: 40×, Scale bar: 200 μm. Data were presented as Means ± SD. ^*^P < 0.05, ^**^P < 0.01.

Nicotine treatment affected the viability of hUC-MSCs in a dose and time-dependent manner. Results from MTT assay showed that the survival of hUC-MSCs treated with nicotine at lower concentrations (≤ 0.35 mg/ml) for the period from 24 to 72 hours was not different from that of the untreated cells. However, when the cells were treated with nicotine at a higher concentration (0.4 mg/ml) for 72 hours, a significant loss in viability of treated cells was observed *in vitro* compared to the untreated cells (*P* < 0.05; Figure 2A, 2B). Cell viability of hUC-MSCs was not significantly impaired until up to concentration of 0.4 mg/ml, so we chose nicotine at the concentrations of 0.1, 0.2 and 0.3 mg/ml for following experiments. Nicotine-treated hUC-MSCs morphology was viewed by microscopy as shown in Figure 2B. The morphology of hUC-MSCs was changed by nicotine in a dose-dependent manner. In nicotine-treated group, the most conspicuous changes were observed in 0.3 mg/ml nicotine treatment. The cells lost cell-cell contact and became round and rough membrane-bearing detached cells, while these changes were absent in untreated cells.

### Nicotine promotes hUC-MSC migration

Transwell migration assays was performed to determine whether nicotine affected the migration potential of hUC-MSCs. The mean numbers of migrated untreated cells in the lower fields after 10 hours were 55.67 ± 9.45, while the mean numbers of hUC-MSCs incubated with nicotine at concentrations of 0.1, 0.2 and 0.3 mg/ml were 92.67 ± 8.50, 142.67 ± 13.01, and 147.00 ± 21.07, respectively. There were significant differences of hUC-MSC migration between the control group and the treatment groups (Figure 2C, 2D).

### Nicotine affects cell cycle and apoptosis in hUC-MSCs

**Figure 3:**
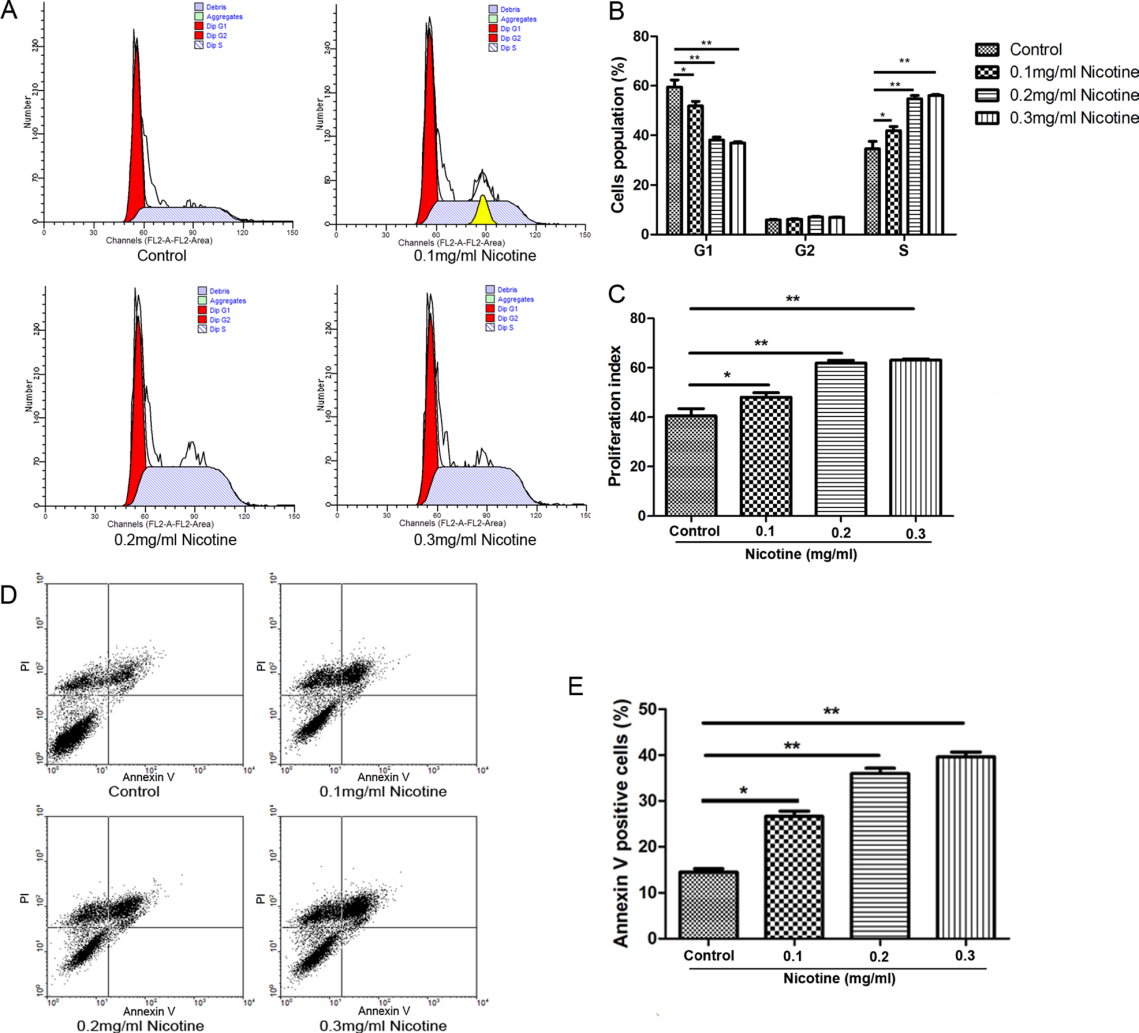
Effect of nicotine on the cell cycle and apoptosis of hUC-MSCs (**A**, **B**) The DNA content analysis indicated the percentage of cells in the S phase were significantly increased compared with the untreated hUC-MSCs. (**C)** PI of nicotine-treated hUC-MSCs were markedly greater than the control group. PI: proliferation index. (**D**) Representative scatter grams from flow cytometry profile represents Annexin V-FITC staining in the X axis and propidium iodide in the Y axis. (**E**) Percentages (%) of Annexin V-positive cells among control or nicotine treated hUC-MSCs. Data were presented as Means ± SD. ^*^P < 0.05, ^**^P < 0.01.

Flow cytometry was used to determine whether nicotine affected hUC-MSC proliferation by interfering cell cycle progression. The proportions of hUC-MSCs in phase S were 34.60 ± 5.19%, 41.91 ± 2.95%, 54.74 ± 2.33% and 56.09 ± 0.72% in cells treated with 0 (control group), 0.1, 0.2 and 0.3 mg/ml of nicotine, respectively (Figure 3A, 3B). In addition, proliferation index (PI) of cells treated with nicotine were markedly greater than the control group (Figure 3C). The results showed that the percentages of cells in S phase were significantly increased in the treated hUC-MSCs compared with the untreated cells. To further investigate the effect of nicotine on hUC-MSC apoptosis, the cells were stained with propidium iodide and Annexin V-FITC and then analyzed by flow cytometry. The proportions of Annexin V-positive cells were 14.50 ± 1.27%, 26.65 ± 1.92%, 35.97 ± 2.02% and 39.62 ± 1.74% in cells treated with nicotine at concentrations of 0, 0.1, 0.2 and 0.3 mg/ml, respectively (Figure 3D, 3E). The results showed that the percentage of hUC-MSCs undergoing apoptosis in the treatment groups were significantly increased compared to that of the control group.

### Nicotine enhances stem cell property in hUC-MSCs

**Figure 4:**
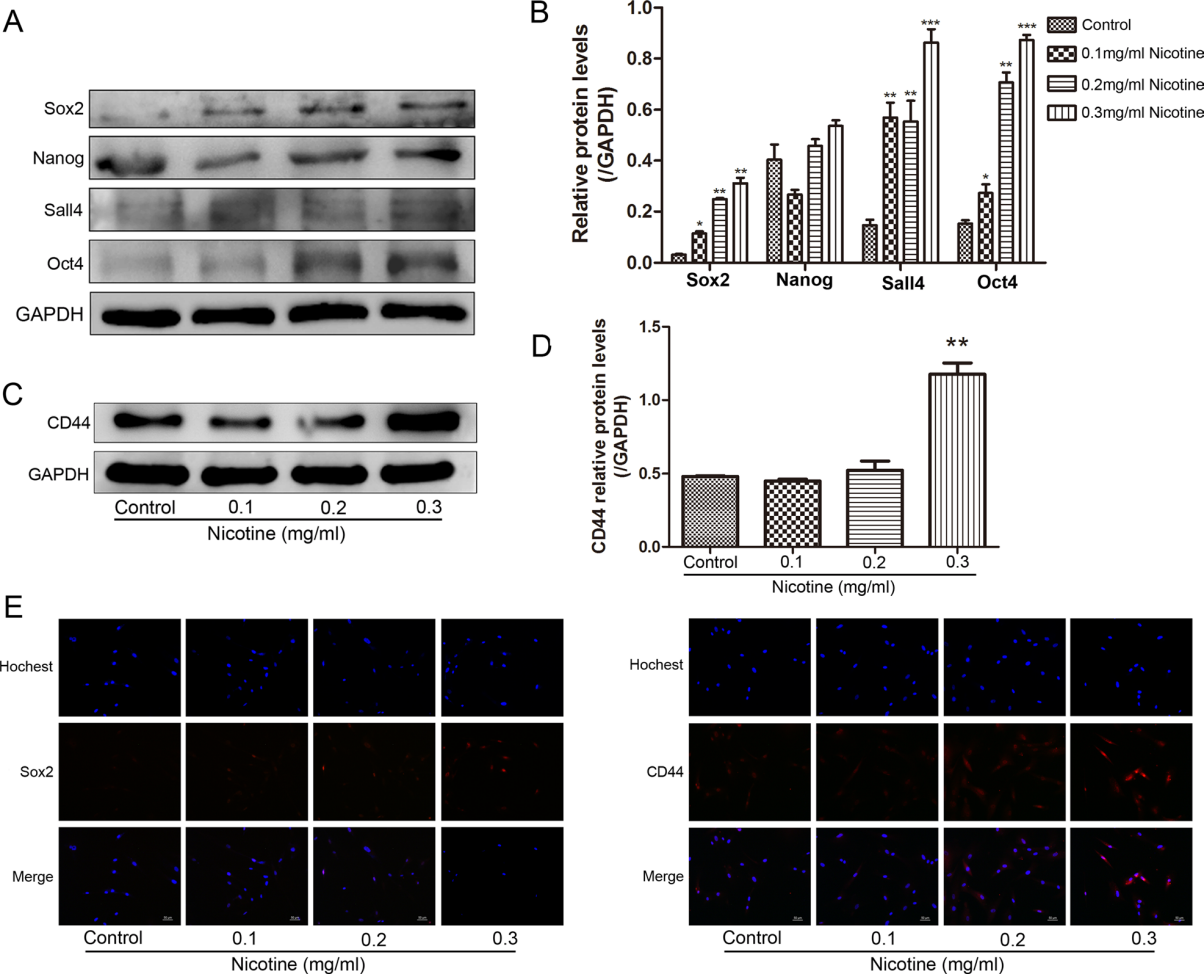
Nicotine induces the acquisition of stemness in hUC-MSCs (**A**, **C**) The expression of Sox2, Nanog, Sall4, Oct4 and CD44 proteins in nicotine-treated hUC-MSCs were determined by western blot. The expression of stem cell markers was upregulated in nicotine treated hUC-MSCs. (**B**, **D**) Three independent experiments were performed to measure the Sox2, Nanog, Sall4, Oct4 and CD44 protein levels. (**E**) Immunofluorescent staining of Sox2 and CD44 in nicotine-treated hUC-MSCs. Immunofluorescence analyses showing increased expression of Sox2 and CD44. Magnification: 200×, Scale bar: 50 μm.

We next investigated whether nicotine played a role in promoting stem cell property in hUC-MSCs. Sox2, CD44, Nanog, Sall4 and Oct4 play essential roles in self-renewal and pluripotency in hUC-MSCs. The western blot was performed to evaluate the expression of these markers between nicotine-treated and untreated hUC-MSCs (Figure 4A–4D). The expression of stem cell markers was upregulated in nicotine-treated hUC-MSCs, indicating enhancement of stem cell property by nicotine. Increased expression of Sox2 and CD44 in nicotine-treated hUC-MSCs was further confirmed by immunofluorescence analysis (Figure 4E).

### Nicotine treatment induces EMT in hUC-MSCs

**Figure 5:**
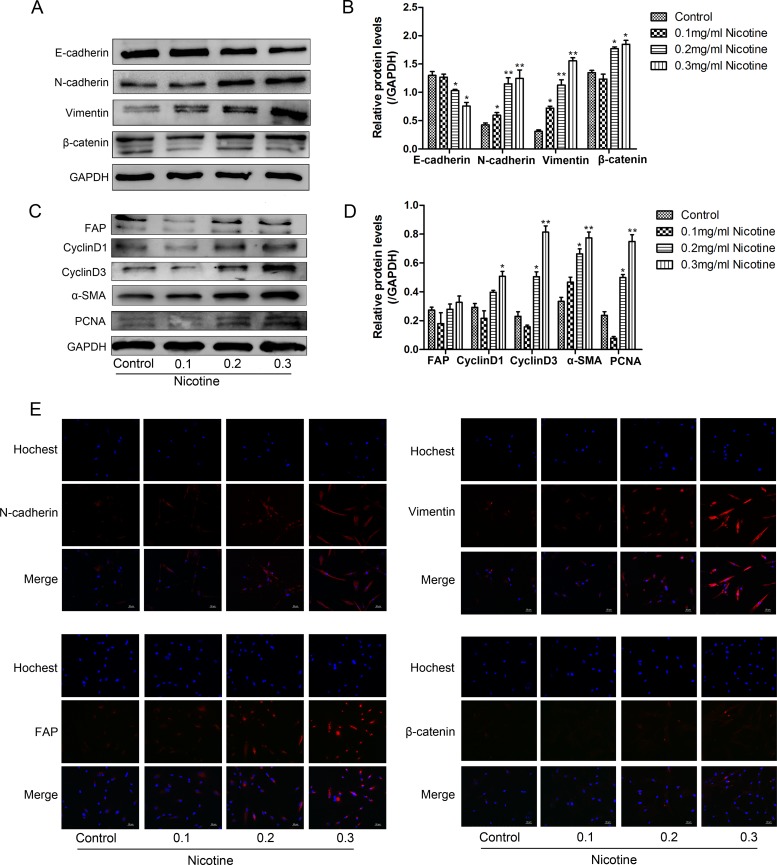
Nicotine induces the acquisition of EMT in hUC-MSCs (**A**, **C**) Western blot analysis showed that nicotine treated cells express markedly higher levels of N-cadherin, Vimentin, β-catenin, FAP, cyclinD1, cyclinD3, α-SMA and PCNA and lower levels of E-cadherin protein. (**B**, **D**) Three independent experiments were performed to measure the EMT markers. (**E**) Immunofluorescent staining of N-cadherin, Vimentin, FAP and β-catenin protein in nicotine-treated hUC-MSCs. These results show that increased expression of N-cadherin, Vimentin, FAP and β-catenin.

Western blot analysis also showed that nicotine-treated hUC-MSCs expressed markedly higher levels of N-cadherin, Vimentin, β-catenin, FAP and α-SMA and lower levels of E-cadherin protein, suggesting that nicotine treatment is capable of inducing EMT (Figure 5A–5D). To further determine the acquisition of EMT phenotype from nicotine treatment, immunofluorescence analysis was performed to confirm the protein levels of N-cadherin, Vimentin, β-catenin and FAP (Figure 5E). The expression of β-catenin and its downstream proteins (cyclinD1, cyclinD3 and N-cadherin) in nicotine-treated hUC-MSCs were increased, suggesting the specific activation of β-catenin signaling by nicotine. PCNA (a marker of tumor proliferation) expression was higher in nicotine-treated cells (Figure 5D), indicating increased proliferation of hUC-MSCs by nicotine.

### Nicotine-treated hUC-MSCs release high level of IL-6

IL-6 is a pleiotropic cytokine that is involved in tumor growth, invasion and metastasis in malignancies. We measured the level of IL-6 released from nicotine-treated hUC-MSCs. The IL-6 level was significantly higher compared to that of untreated hUC-MSCs (Figure 7E), indicating that nicotine-treated hUC-MSCs might facilitate the malignant progression and metastasis by secretion of IL-6.

**Figure 6:**
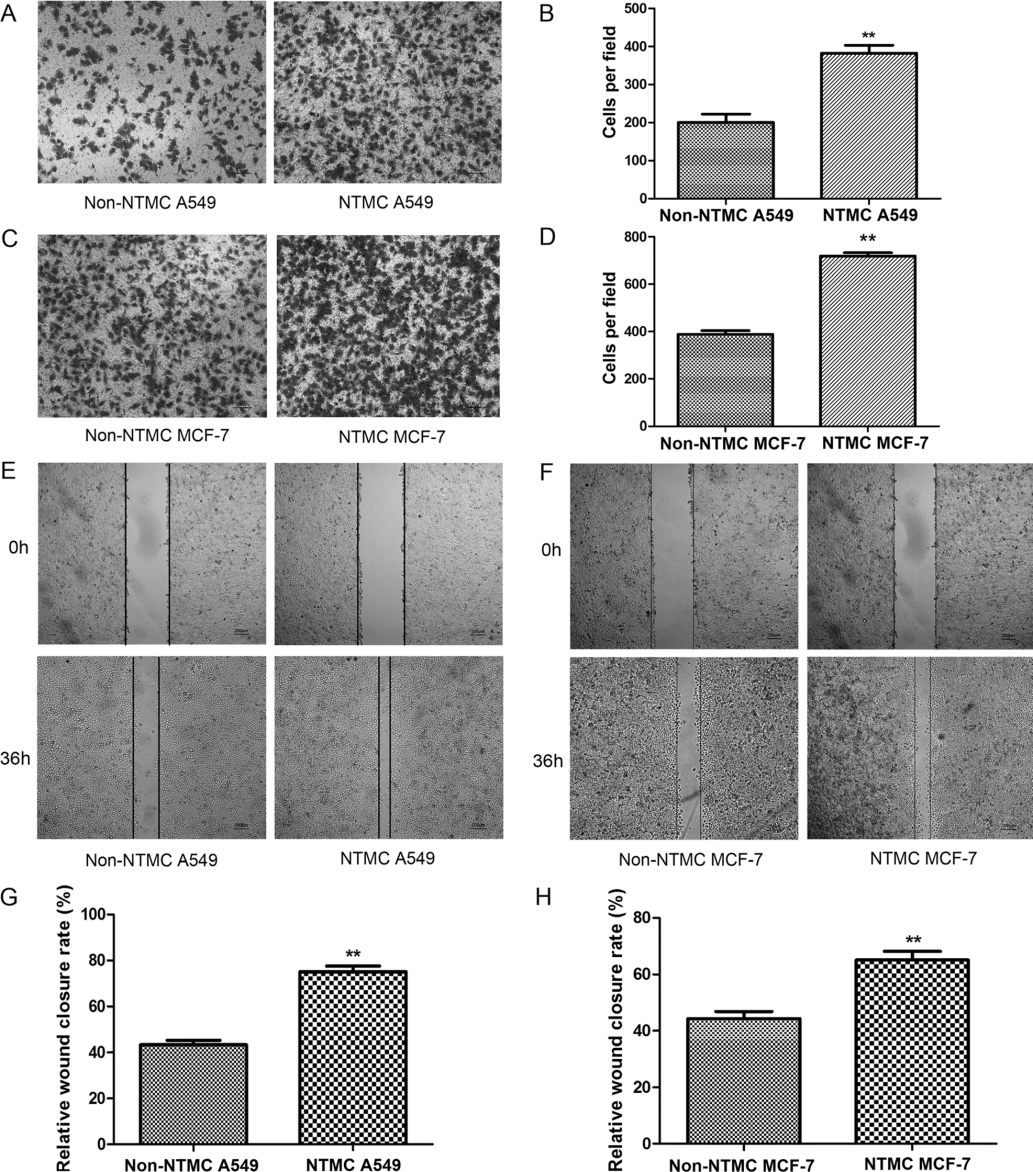
Nicotine-treated hUC-MSCs promote the migration of A549 cells and MCF-7 cells (**A**, **C**) Representative images of transwell migration assay in A549 cells (A) and MCF-7 cells (C). Magnifications: 100×, Scale bar: 100 μm. (**B**, **D)** Histogram of the number of migrated A549 cells (B) and MCF-7 cells (D). (**E, F**). Scratch wound assay results. Example images of wound closure in the control and NTMC A549 cells (E) and NTMC MCF-7 cells (F) Magnifications: 40×, Scale bar: 200 μm. (**G**, **H**) Wound closure ratios in the control and NTMC A549 cells (G) and NTMC MCF-7 cells (H). NTMC: 0.3 mg/ml nicotine-treated hUC-MSCs co-cultured. There were significant differences between the experimental group and the control group. Data were presented as Means ± SD. ^**^P < 0.01.

**Figure 7:**
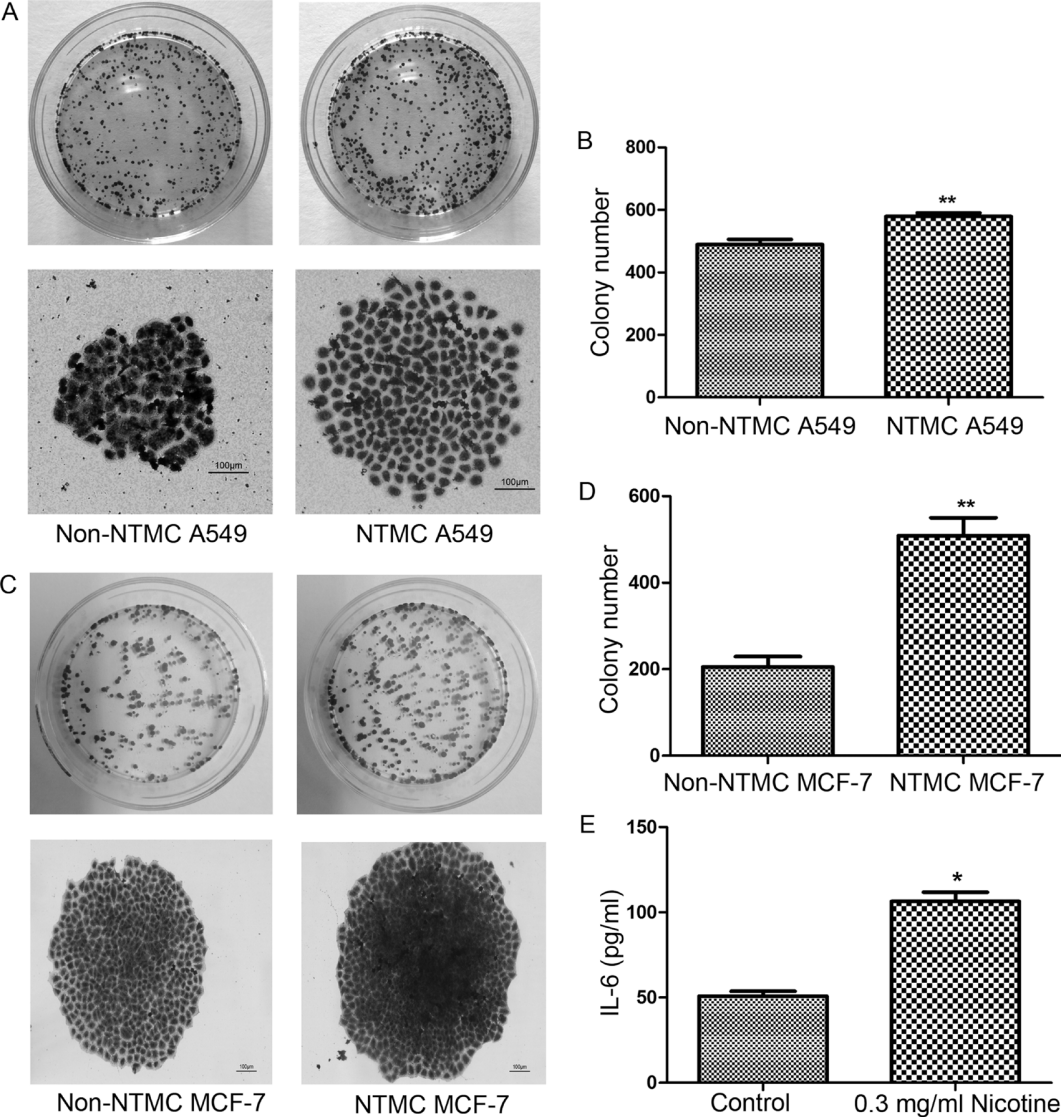
Effect of nicotine-treated hUC-MSCs on colony formation of A549 cells and MCF-7 cells (**A**, **C**) Representative images of cell colony formation assay in A549 cells (A) and MCF-7 cells (C) Colony formation assays revealed that the number of cell colonies in the NTMC group were higher than that in the non-NTMC group and NTMC group formed spheres larger in diameter. Magnifications: 100×, Scale bar: 100 μm. (**B**, **D)** Histogram of the number of colonies in A549 cells (B) and MCF-7 cells (D) (**E**) ELISA analysis of supernatant content. The IL-6 level was significantly high compared to that of control group. Data were listed as mean ± SD of three wells. Data were presented as Means ± SD. ^**^P < 0.01.

### Nicotine-treated hUC-MSCs promote the migration of A549 cells and MCF-7 cells

We treated hUC-MSCs with 0.3 mg/ml nicotine for 24 hours and then cocultured them with human non-small-cell lung cancer A549 cells and human breast cancer MCF-7 cells in a cell-cell contact-independent manner. We evaluated the migration of A549 cells and MCF-7 cells isolated from the pre-coculture with nicotine-treated hUC-MSCs (here named NTMC). In the transwell migration assays, the mean numbers of migrated A549 cells without pre-coculture with nicotine-treated hUC-MSCs (here named non-NTMC) after 10 hours were 200.00 ± 38.43, whereas the mean numbers of NTMC A549 cells were 382.33 ± 26.23. The mean numbers of migrated non-NTMC MCF-7 cells were 387.67 ± 27.54, whereas the mean numbers of NTMC MCF-7 cells were 719.67 ± 24.58. The migrated cells were significantly increased in NTMC A549 and NTMC MCF-7 cells compared with the non-NTMC A549 cells and non-NTMC MCF-7 cells (Figure 6A–6D). Scratch wounds were inflicted in NTMC A549 cells and NTMC MCF-7 cells and differences were observed between the groups after 36 hours of treatment (Figure 6E–6H). The wound closure rates for A549 cells were 43.32 ± 3.23% and 75.11 ± 4.19% in the non-NTMC and NTMC group, respectively. The wound closure rates for MCF-7 cells were 44.38 ± 4.35% and 65.08 ± 5.36% in the non-NTMC and NTMC group, respectively. There were significant differences in migration and would closure rates of both A549 and MCF-7 cells between the NTMC and non-NTMC groups (*p* < 0.01). These results indicate that NTMC A549 cells and NTMC MCF-7 cells may have higher metastatic potential.

### Nicotine-treated hUC-MSCs enhance the colony formation

Compared to non-NTMC A549 cells and non-NTMC MCF-7 cells, colony formation of NTMC A549 cells and NTMC MCF-7 cells were significantly increased (*P* < 0.01). In addition, NTMC A549 cells and NTMC MCF-7 cells formed spheres larger in diameter compared to non-NTMC cells (Figure 7A–7D) (*P* < 0.01). These data demonstrated that nicotine-treated hUC-MSCs enhance colony formation of A549 and MCF-7 cells.

### Nicotine-treated hUC-MSCs enhance tumor growth *in vivo*

**Figure 8:**
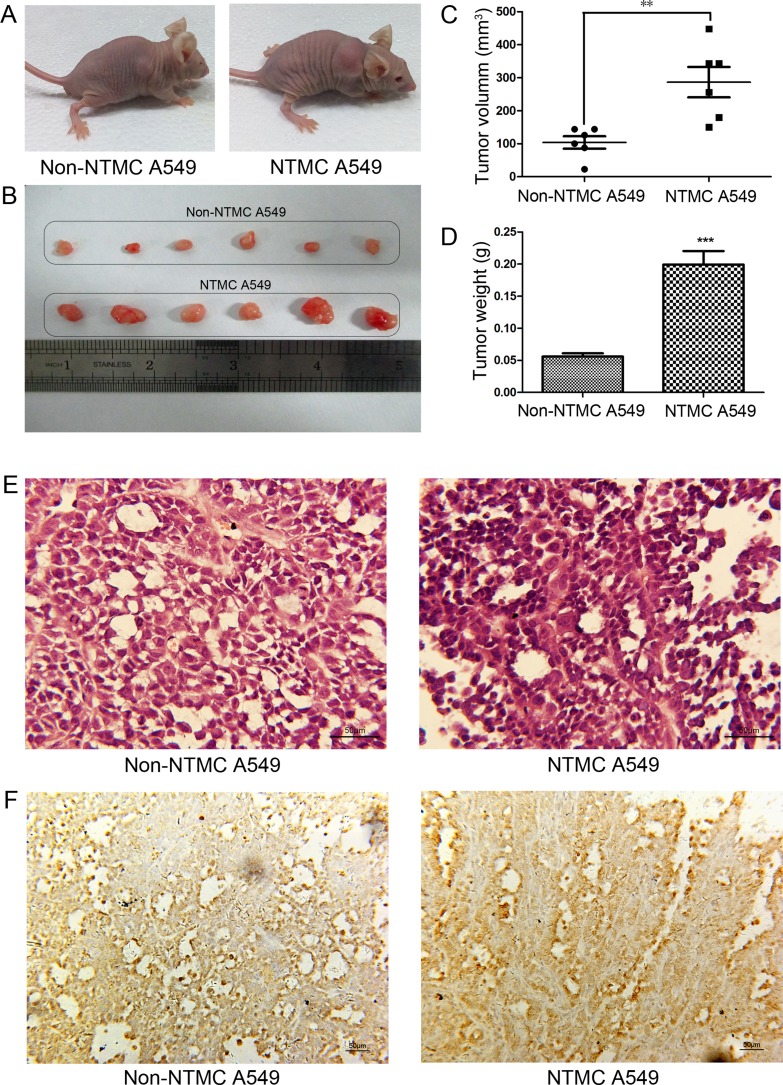
Nicotine-treated hUC-MSCs enhanced growth of A549 cells *in vivo* (**A)** The representative images of tumor-bearing mice. (**B)** Tumor tissues were photographed 20 days after tumor cell inoculation. (**C**, **D**) The weight and volume of tumor issues removed from experimental and control groups. (**E)** Histologic outcomes of H&E staining, magnification, 400×, Scale bar: 50 μm. (**F)** The expression of PCNA were evaluated by immunohistochemical in tumor tissues. magnification, 200×, Scale bar: 50 μm. Data were presented as Means ± SD. ^**^P < 0.01, ^***^P < 0.001.

To investigate whether nicotine-treated hUC-MSCs could promote tumor growth *in vivo*. We used NTMC A549 cells to establish xenograft tumors in nude mice. The images of tumor-bearing mice were shown in Figure 8A. In the NTMC group, tumor nodules started to form at day 5 after injection while it was not observed in the non-NTMC group. As shown in Figure 8B–8D, the tumor weight and volume in NTMC group were increased compared to that in the non-NTMC group. In addition, the neoplasm tissues presented high heterogeneity, abnormally elevated nuclear/cytoplasmic ratios, and derangement distribution in some regions (Figure 8E). PCNA expression was higher in tumors established from NTMC A549 cells than that in non-NTMC group (Figure 8F). Taken together, these results suggested that nicotine-treated hUC-MSCs can promote tumor growth *in vivo*.

## DISCUSSION

NSCLC is the leading cause of death among human malignancies worldwide, with over a million deaths annually [43, 44]. Despite intensive efforts were imposed on treatment practices, the survival rate for lung cancer has not been improved substantially in the past years. Cigarette smoking is a well-known risk factor in lung cancer and nicotine is the major chemical component in cigarette smoke. It is well known that tumor cells secrete chemokines, cytokines and growth factors that are able to recruit and activate MSCs which can migrate to the tumor site of numerous types of cancers and play a key role in cancer progression *in vivo* [45, 46]. In the present study, we found that nicotine-enhanced stemness and EMT of hUC-MSCs promote tumor formation and growth *in vivo*.

Tsz Kin *et al.* demonstrated that cigarette smoking hindered proliferation, migration and differentiation potentials of human periodontal ligament-derived stem cell (PDLSC) and further provided the evidence that miRNAs were an essential regulator in these cigarette smoking-associated functional changes [47]. Here we hypothesize that nicotine may also have an impact on hUC-MSCs and indeed demonstrated the effects of nicotine on the biological characteristics of hUC-MSCs, especially their potentials related to tumor formation and cancer progression.

MSCs that we isolated and cultured were characterized with surface markers of hUC-MSCs and without surface markers of lymphocytes. When cultured in appropriate osteogenic or adipogenic differentiation medium, these cells were capable of differentiating into osteocytes and adipocytes, respectively. Therefore, these cells with capability of self-renewal and multilingeage differentiation, were designated as hUC-MSCs. In this study, we demonstrated the effects of nicotine on cell proliferation of hUC-MSCs. Nicotine at concentrations ranging from 0.08 mg/ml to 0.35 mg/ml did not have any significant effects on cell proliferation of hUC-MSCs. However, the proliferation decreased significantly after treatment with nicotine at 0.4 mg/ml. In addition, we observed vacuolization only in hUC-MSCs cultured with 0.3 mg/ml nicotine (Figure 2). The vacuolization may be attributed to the uptake and accumulation of nicotine in the cell membrane. The changes of cell morphology of hUC-MSCs in response to nicotine in a dose-dependent manner are suggestive of functional changes. Cell morphology of hUC-MSCs is directly related to their adhesion, extension, migration, and proliferative capabilities [48]. It has been reported that nicotine attenuates cell proliferation of human alveolar bone marrow-derived MSC [49] and PDL fibroblasts [50]. In addition, nicotine induces apoptosis in PDLSC [51]. Our study also proved that nicotine inhibits cell proliferation of hUC-MSCs (Figure 2, Figure 3). Several previous studies showed that nicotine inhibits the migration of human MSCs [52] and PDL fibroblast [53]. However, here we found that nicotine increases the migration of hUC-MSCs. These discrepancies in the effects of nicotine on cell migration may be due to the bimodal effects of nicotine at different concentrations [54].

EMT is considered as one of the major mechanisms involved in tumor metastasis. Previous studies suggest that cells undergoing EMT acquire stem-cell properties [55, 56]. These stem cell-like cells in tumors are described as CSCs. CSCs have been characterized with different specific cell surface markers such as Sox2 and Nanog, the major stemness-related genes, and have been considered as the key factor for tumor initiation and progression [57–59]. We hypothesized that nicotine might endow hUC-MSCs with carcinogenic features. To test this hypothesis, we first examined the phenotype of nicotine-treated hUC-MSCs *in vitro*. The results showed that nicotine-treated hUC-MSCs not only displayed a morphological shift from epithelial to fibroblastic phenotype, but also presented the decrease of E-cadherin protein expression, increased Vimentin, N-cadherin and β-catenin protein expression. In addition, we showed that the activated hUC-MSCs, which were cocultured with nicotine, markedly enhanced the protein expression of Sox2, CD44, Nanog, Sall4 and Oct4. The upregulation of these stemness-related factors indicated the acquirement of stem-cell properties in nicotine-treated hUC-MSCs. To understand the impact of nicotine-treated hUC-MSCs on tumor formation and progression of tumor cells, our results showed markedly increased mono-colony forming and migration *in vitro* in not only NTMC A549 cells but also NTMC MCF-7 cells. Furthermore, nicotine-treated hUC-MSCs elevated tumorigenicity of A549 cells *in vivo.* These results suggest that nicotine-treated hUC-MSCs have general effects on tumor formation and growth for various cancers.

It is possible that nicotine-treated hUC-MSCs may work through inflammatory pathways to promote the malignancy of tumor cells. Takahashi et al described a model of lung tumorigenesis in which tobacco smoke acts as a tumor promoter, causing increased proliferation of chemically and genetically induced lung cancer cells *in vivo* through IKKβ- and JNK1- mediated inflammatory signaling [60]. IL-6 can promote tumorigenicity, angiogenesis and metastasis [61, 62]. In addition, IL-6 has been shown to be a direct regulator for self-renewal of breast cancer cells through signal transducer and activator of transcription 3 (STAT3) activation mediated by the IL-6 receptor/GP130 complex [63]. Generally, the biological functions of IL-6 are achieved via the Janus kinases/STAT3 (JAK/STAT3) pathway that is frequently presented in cancers including lung cancer. JAK/STAT3 pathway is widely involved in transformation, EMT, tumorigenicity and metastasis [64–68]. Here we observed that the IL-6 levels were extremely high in nicotine-treated hUC-MSCs compared to untreated hUC-MSCs (Figure 7E), indicating that IL-6 might be involved in driving lung cancer cells and breast cancer cells into more aggressive and invasive phenotypes after coculture with nicotine-treated hUC-MSCs and promoting tumor formation and growth. These findings provide a mechanistic link between nicotine-treated hUC-MSCs and IL-6 for the progression of tumor malignancy in non-small cell lung cancer.

## MATERIALS AND METHODS

### Isolation and culture of hUC-MSCs

In our previous study, we had successfully isolated MSCs from the human umbilical cord [69, 70]. Fresh umbilical cords were collected from healthy donors with informed consents at the First People’s Hospital of Zhenjiang, China. We used those hUC-MSCs for the current set of experiments. The cells of passages 3 to 5 were used in the experiments. HUC-MSCs were plated at a density of 1 × 10^5^ cells per 25 cm^2^ flask (Coning, USA). The cells were cultured in Dulbecco’s modified Eagle’s medium with low glucose (L-DMEM) containing 10% fetal bovine serum (FBS) (Gibco, Carlsbad, CA, USA) and 1% antibiotics (100 U/ml penicillin and 100 U/ml streptomycin; Beyotime, Haimen, China) in a humidified atmosphere with 5% CO_2_ at 37°C, and the medium was changed every 3 days thereafter. When the adherent cells reached nearly 80% to 90% confluence, the cells were digested by 0.25% trypsin-EDTA (Invitrogen, USA) and subcultured in a new flask for further expansion.

### Flow cytometry

The phenotype of hUC-MSCs was analyzed by flow cytometry using a BD Accuri C6 flow cytometer (Becton Dickinson, San Jose, CA, USA). The third passage cells were analyzed for expression of hUC-MSCs markers. The cells were stained with phycoerythrin (PE)-labled anti-CD29, anti-CD90, anti-CD105 and fluorescein isothiocyanate (FITC)-labled anti-CD19 (Becton Dickinson, USA). Mouse immunoglobulin G (IgG) was used as the negative control for each treatment. Then the cells were washed twice with phosphate-buffered saline (PBS) and fluorescence measured with flow cytometry.

### Multi-differentiation capacity analysis

### Osteogenesis

HUC-MSCs were cultured with osteogenic differentiation medium (0.1 μM dexamethasone, 10 mM β-glycerophosphate, 4 μg/ml basic fibroblast growth factor (bFGF) and 50 μg/ml ascorbic acid; Sigma-Aldrich, MO, USA) for 2 weeks. At the end of induction, the cells were stained with alkaline phosphatase (ALP) based on the manufacturer’s instructions (Beyotime, Haimen, China).

### Adipogenesis

The cells were plated in 24-well plates in L-DMEM containing 10% FBS. When the cells reached 60 to 80% confluence, the medium was changed into adipogenic differentiation medium (10 μg/ml insulin, 0.5 mM IBMX, 200 μM indomethacin and 1 μM dexamethasone; Cyagen, Guangzhou, China) for 3 days and switched to maintenance medium (supplemented with 10% FBS and 10 μg/ml insulin; Cyagen, Guangzhou, China) for 1 day. After adipogenic differentiation of 3 weeks, to investigate the capability of hUC-MSCs to induce adipogenesis, lipid droplets were stained with Oil Red O.

### Reverse transcription polymerase chain reaction (RT-PCR)

Total RNA was extracted with TRIZOL Reagent (Invitrogen Life Technologies, Carlsbad, CA, USA) following the manufacturer’s instructions. cDNA was synthesized with Superscript II reverse transcriptase using Oligo (dT) primer (Toyobo, Osaka, Japan) according to the manufacturer’s protocol. The PCR amplification was performed for 30 to 35 cycles using an ABI 2720 thermal cycler (Applied Biosystems). PCR System using the following program: 94°C for 5 minutes, 94°C for 30 seconds, annealing at 57-70°C for 30 seconds (Supplementary Table 1 for temperatures used), 72°C for 30 seconds and a final extension at 72°C for 10 minutes. The PCR products were separated on a 1.5% agarose gel, with ethidium bromide staining, and visualized under UV light on a UV transilluminator. GAPDH gene was used as an internal control. Primer sequences are listed in Supplementary Table 1.

### Cell proliferation assay

The proliferation of hUC-MSCs was evaluated by MTT (3-(4,5-dimethylthiazol-2-yl)-2,5-diphenyltetrazolium bromide; Amresco, USA) assay. The cells (2 × 10^3^) were seeded on 96-well plates with L-DMEM containing 10% FBS, and allowed to attach overnight. Then the cells were treated with or without nicotine at various concentration (0.08 to 0.4 mg/ml) for 24, 48, and 72 hours. MTT was added in each well and incubated at 37°C for 4 hours. Then the medium was discarded and 150 μl dimethyl sulfoxide (DMSO) was added to each well for 10 minutes to ensure complete solubilization of the purple formazan crystals. The absorbance of the solution was measured using an enzyme-linked immunosorbent assay reader at 490 nm (FLX800, Biotek).

### Observation of cell morphology

In order to assess the effects of nicotine on cell morphology, hUC-MSCs were seeded on 6-well plates with L-DMEM containing 10% FBS. After overnight incubation, the cells treated with or without nicotine at concentrations of 0.1, 0.2 and 0.3 mg/ml for 24 hours. The cell images were analyzed using bright field microscope under 40× magnifications (Ti-S, Nikon Corporation, Tokyo, Japan).

### Cell migration analysis

Migration analysis were performed based on the manufacturer’s guidelines (Corning Inc, Corning, NY, USA). HUC-MSCs were treated with or without nicotine at concentrations of 0.1, 0.2 and 0.3 mg/ml for 24 hours. In addition, a coculture system was established by culturing hUC-MSCs in the Transwell insert with A549 cells or MCF-7 cells in the plastic plates (3412, Transwell, Coning, USA). We examined A549 cells and MCF-7 cells cocultured with hUC-MSCs which were treated with 0.3 mg/ml nicotine for 24 hours. A549 cells and MCF-7 cells were pre-cocultured with nicotine-treated hUC-MSCs in a cell-cell contact-independent manner (here named NTMC). Nicotine-treated hUC-MSCs, NTMC A549 cells and NTMC MCF-7 cells were resuspended in 200 μl serum-free medium and seeded into the upper chamber at 1 × 10^5^ cells per well, respectively. The lower chamber was filled with 800 μl complete medium. After incubation at 37°C for 10 hours, the upper cells remaining in the upper membrane were wiped off smoothly, while the cells migrated to the lower surface of the membrane were fixed with 4% paraformaldehyde for 30 minutes and stained with crystal violet for 30 minutes. Three random fields (magnifications, 100×) were observed under the microscope.

### Cell cycle assay

HUC-MSCs were seeded on 6-well plates for each data point and cultured overnight. After overnight incubation, the cells were treated with nicotine (0.1, 0.2 and 0.3 mg/ml) for additional 24 hours. Then, the cells were collected and washed twice with PBS and stained with propidium iodide (PI; Sigma-Aldrich, St. Louis, MO, USA) for 30 minutes at 4°C in dark condition. The stained cells were analyzed by flow cytometry using a BD Accuri C6 flow cytometer (Becton Dickinson). Proliferation index (PI ) = (S + G2)/(G1 + S + G2).

### Apoptosis assay

Apoptotic cells were detected with Annexin V/Dead Cell Apoptosis Kit (Invitrogen, Carlsbad, CA, USA) according to manufacturer’s instructions. HUC-MSCs were exposed to nicotine at concentrations of 0.1, 0.2, 0.3 mg/ml for 24 h. The cells then were collected and washed twice with PBS and stained with Annexin V-FITC and PI for 30 minutes in the dark. The stained cells were analyzed by flow cytometry using a BD Accuri C6 flow cytometer (Becton Dickinson).

### Protein extraction and western blot analysis

When hUC-MSCs reached 80% confluence, the medium was changed to maintain a constant level of nicotine (0.1, 0.2 and 0.3 mg/ml) for 24 hours. Nicotine-treated hUC-MSCs were plated in complete medium and grown as normal until they reached 90% confluence. Then the total cellular protein of the cells was extracted using RIPA lysis buffer (Vazyme Biotech, China) and phenylmethanesulfonyl fluoride (PMSF; Beyotime, Haimen, China). Cell lysates were separated on a 10% sodium dodecyl sulfate polyacrylamide gel electrophoresis (SDS-PAGE, Beyotime) and then transferred onto polyvinylidene fluoride (PVDF) membrane (Beyotime). The membranes were blocked with 5% skim milk in TBS-T (20 mM Tris-HCl, 150 mM NaCl, and 0.1% Tween-20) at room temperature for 2 hours and incubated with primary antibodies at incubated with primary and horseradish peroxidase-conjugated secondary antibodies (goat anti-rabbit or goat anti-mouse antibodies; diluted at 1:2,000; CWBIO) and detected by the Amersham ECL detection system (GE Healthcare Life Sciences, Little Chalfont, U.K). Primary antibodies used in the experiments were as follows: E-cadherin (1:200; Santa Cruz Biotechnology, Dallas, TX), N-cadherin (1:400), Vimentin (1:300), β-catenin (1:500), Sox2 (1:200), Nanog (1:800), Sall4 (1:600), Oct4 (1:300), Cyclin D1 (1:200) and Cyclin D3 (1:200), α-smooth muscle actin (α-SMA; 1:1000), proliferating cell nuclear antigen (PCNA; 1:500), fibroblast activation protein (FAP; 1:500), CD44 (1:500) (Bioworld Technology, St. Louis Park, MN) and GAPDH (1:1500; Cell Signaling Technology). GAPDH was used as the loading control.

### Immunofluorescence analysis

Nicotine-treated hUC-MSCs were fixed in 4% paraformaldehyde for 30 minutes, permeabilized for 10 minutes with 0.1% Triton-X 100 blocked with 5% bovine serum albumin (BSA), and incubated with rabbit monoclonal rabbit monoclonal anti-N-cadherin (1:200), Vimentin (1:100), β-catenin (1:200) and FAP (1:500) (Bioworld Technology) antibodies overnight at 4°C, followed by incubation with Cy3-labeled anti-rabbit IgG secondary antibody (1:800) at 37°C for 45 minutes in the dark. Finally, the nuclei were counterstained with Hoechst 33342 (1:200; Sigma-Aldrich). Fluorescent images were acquired at 200× using an inverted fluorescence microscope (Ti-S, Nikon).

### Scratch wound assay

NTMC A549 cells and NTMC MCF-7 cells were allowed to grow until 80% confluency and scratched vertically with a 0.2-ml sterile pipette tip. The resulting debris was removed by gentle washing with PBS. Subsequently the wound healing was acquired with an inverted microscope (Ti-S, Nikon) and photographed at pre-selected time points (36 hours) in three randomly selected fields for each condition and time point.

### Colony forming assay

NTMC A549 cells and NTMC MCF-7 cells were plated at a density of 500 cells per plate in a 6-well plate. After culturing for 12 days, the colony units were fixed with 4% paraformaldehyde, stained with crystal violet for 30 minutes and then photographed and counted. The experiment was performed in triplicate.

### Cytokine detection in the supernatant

HUC-MSCs were treated with nicotine (0.3mg/ml) for 24 h. The supernatant then were collected. The expression levels of IL-6 in each group were quantified using ELISA kits (BD Biosciences Pharmingen, San Diego, CA, USA), according to the manufacturer’s instructions. The experiments were performed in triplicate.

### Animal model

All the animal practiced in this study were performed under approval of the University Committee on Use and Care of Animals of Jiangsu University. A549 cells were cultured with hUC-MSCs *in vitro* in transwell without cell-cell contact and isolated and then injected subcutaneously into the flank of 6-week-old BALB/c nude mice (*n* = 6 for each group). All tumors were surgically resected 20 days after injection, photographed and weighted. Tumor volume (TV) was calculated by caliper measurement and calculated by the formula: TV (mm^3^) = (length × width^2^)/2.

### Immunohistochemistry

The protein levels of PCNA in formalin-fixed paraffin-embedded tissues and were detected by immunohistochemistry. Briefly, the tissue slides were incubated with primary antibody and secondary antibody (Bioworld Technology), visualized with chromogen 3,3′-Diaminobenzidine (DAB) and then hematoxylin was applied for counter-staining. Tissues sections were chosen randomly and counted under a microscope at least six fields for each tissue.

### Statistical analysis

The statistically significant differences between groups were assessed by analysis of variance (ANOVA) or *t* test using the GraphPad Prism V 5.0 software program (GraphPad, San Diego, CA, USA). The results are expressed as the means ± SD from three different replicates from individual assays. *P* value < 0.05 was considered statistically significant.

## SUPPLEMENTARY MATERIALS TABLE


