# Supplementary material for: Nicotine-enhanced stemness and epithelial-mesenchymal transition of human umbilical cord mesenchymal stem cells promote tumor formation and growth in nude mice
Source: Oncotarget. 2017 Nov 27;9(1):591–606. doi: 10.18632/oncotarget.22712 (PMC5787492; doi:10.18632/oncotarget.22712)
Supplement: Supplementary file 1 [file oncotarget-09-591-s001.pdf]

Nicotine-enhanced stemness and epithelial-mesenchymal transition of human umbilical cord mesenchymal stem cells promote tumor formation and growth in nude mice

SUPPLEMENTARY MATERIALS

Table 1: List of primers

Gene	Primers sequence (5′ to 3′)	Size (bp)	Annealing (°C)
BMP-3-F	GACCCTCCAATCCAACCA	287	60.5
BMP-3-R	ACGCTTTCAGGCTCACAA	287	60.5
PPARγ-2-F	TGAAG ACTCATGTCTCTC	320	58.0
PPARγ-2-R	GGATTTGGTCGTATTGGG	320	58.0
GAPDH-F	GAGTCTACTGGCGTCTTCAC	272	58.0
GAPDH-R	GTCTTCTGAGTGGCAGTGAT	272	58.0
